# Sexual maturation of HIV-infected and uninfected male children in Abakaliki, South-East, Nigeria: a cross-sectional study

**DOI:** 10.11604/pamj.2022.42.133.29305

**Published:** 2022-06-17

**Authors:** Chijioke Ogodo Ogeh, Maria-Lauretta Orji, Kenechukwu Kosisochukwu Iloh, Chijioke Joel Nweke, Blessed Uzochukwuamaka Ogeh, Ogochukwu Nneka Iloh, Ifeoma Josephine Emodi

**Affiliations:** 1 Alex-Ekwueme Federal University Teaching Hospital, Abakaliki, Nigeria,; 2 Department of Paediatrics, University of Nigeria Teaching Hospital, Ituku-Ozalla, Enugu, Nigeria,; 3 Alex-Ekwueme Federal University, Ndufu-Aleke Ikwo, Ebonyi State, Nigeria

**Keywords:** Sexual maturation, adolescents, males, HIV-infected

## Abstract

**Introduction:**

HIV infection, through various mechanisms causes a derangement in sexual maturation. This study compared the Marshal and Tanner staging of HIV-infected and uninfected males. The aim of the study was to determine the sexual maturation in male children infected with HIV on HAART in Abakaliki.

**Methods:**

this was a cross-sectional and comparative study involving 80 HIV-infected boys aged 8-17 years and 80 uninfected counterparts matched for age and socio-economic class. Stages of sexual maturation (testicular size and pubic hair) were determined according to the method proposed by Marshall and Tanner. The testicular size was measured using an orchidometer. Data analysis was done with SPSS version 20. Structured questionnaire was used to collect information on socio-demographics.

**Results:**

assessment of pubic hair development, showed that 45 (56.2%) of the subjects were in the pre-pubertal stage compared to 27 (33.8%) among the controls, this relationship was statistically significant (p=0.005, OR = 2.5, C.I=1.3-4.8). The mean testicular volume among subjects was found to be 8.29 ± 8.26mls compared to 11.57 ± 8.26mls found in controls. This relationship was also statistically significant. There were significant statistical relationships between duration on HAART and clinical stages of disease with both pubic hair development and testicular volume of subjects and controls.

**Conclusion:**

HIV-infected males had significantly delayed onset and progression of sexual maturation. Routine assessment of the sexual maturation of HIV-infected children as well as addressing the modifiable variables influencing sexual maturity is recommended.

## Introduction

Amongst all the developmental changes that occur in adolescence, sexual maturation is the most significant in its influence on the behaviour of boys and girls [1]. The physical changes that occur during the development of secondary sexual characteristics were staged by Marshall and Tanner [2]. They devised a method of classifying the adolescent based on the level of sexual maturation into five stages [2]. This classification utilizes size of the scrotum and testes, and the penile shafts as well as the pattern of distribution and quantity of the pubic hair [2]. The age at initiation, sequence and period of puberty in males differ among persons and between children from different ethnic groups, countries, and races and also differ among developed and developing nations [3]. Normal initiation of sexual maturation in males may range from nine years to 14 years and may take an average of 1.5-6 years to be completed [3]. The earliest age at which the physical signs of puberty occurs is 8 years (SMR 2) in females and 9.5 years (SMR 2) in males [4]. Factors that influence sexual maturation could be hormonal, physical activity level, chromosomal, ethnic, constitutional, socio-economic status, nutritional and chronic illnesses like HIV [5-7]. Hormonal control of sexual maturation depends primarily on the activation of the pituitary gland with subsequent release of luteinizing hormone (LH) and follicle-stimulating hormone (FSH) [8]. HIV infection may affect metabolic and endocrine functions and thus alter hormonal systems involved in the control of growth and development [9]. Reported endocrine problems related to HIV infection in children include growth deceleration and delayed onset of puberty [10]. A perceived or actual delay in sexual maturation and biological development can lead to the development of poor body image and low self-esteem which may result in psychosocial problems such as eating disorders, depression, social withdrawal, isolation, declining academic performance, school avoidance, teasing and bullying by peers [11].

Assessment of sexual maturation using testicular size and pubic hair development in boys can be done by two methods, namely; the adolescent self-report and the physician assessment [12]. The disadvantage of adolescent self-report method is that a bias could occur resulting in over or under reporting. The self-report method is also easier to perform and subjects are not exposed to physical examinations [12]. In the physician assessment method, the physician physically examines the respondents to decide their maturation stage and grades them according to the criteria of Tanner [12]. This study therefore, aimed to compare the sexual maturation of perinatally HIV-infected males aged 8-17 years and their uninfected counterparts at the Alex Ekwueme Federal University Teaching Hospital and Mile Four Mission Hospital, Abakaliki, Ebonyi State, Nigeria.

## Methods

The study was conducted at the Alex Ekwueme Federal University Teaching Hospital and Mile Four Mission Hospital, Abakaliki, Ebonyi State, Nigeria using physician assessment.

**Study site:** the study was carried out in the paediatric HIV clinics of both hospitals. The clinics run once a week in each hospital and has an average attendance of 20 and 15 patients respectively. A total of 200 children were registered in the paediatric HIV clinic, (105 males were aged between 8 and 17 years) as at August 2018 from both hospitals. The controls were recruited from the Children Outpatient Clinic (CHOP). This outpatient clinic runs daily, except on weekends with an average attendance of 350 patients per week.

**Study design:** this was a cross-sectional and comparative study. HIV-infected males aged 8-17 years who met the inclusion criteria were consecutively enrolled from the HIV clinic until the desired sample size was achieved.

**Study population:** the study subjects were HIV-infected males aged 8-17 years who were born to women with documented HIV-1 infection during pregnancy or at the time of delivery and in whom HIV infection had been diagnosed through detection of viral markers (DNA PCR) or the persistence of HIV-1 antibodies (investigated by means of enzyme linked immunosorbent assay) after 18 months of life. The controls were HIV-uninfected males who were matched for age and social class and without chronic diseases such as sickle cell anemia, chronic kidney disease or asthma attending the children outpatient clinic.

**Sample size determination:** the sample size required was determined using the method as shown:


$$Sample\;Size\;(n)=\frac{r+1}{1}\times \frac{(p*)(1-P*){\left(Z_{\beta }+Z_{\alpha /2}\right)}^{2}}{{\left(p_{1}-p_{2}\right)}^{2}}$$

Where r = ratio of control to cases (subjects), 1 for equal number of cases and control.


$$p*=\frac{average\;proportion\;\exp osed\;proportion\;of\;\exp osed\;cases\;+proportion\;of\;control\;\exp osed}{2}$$

Fifty percent (50% (0.5)) was used when there was no recognizable estimate. Z_β_= normal standard variate for significant level as identified in previous section. i.e normal standard variate for power of 80% which is equal to 0.84. Z_α/2_= normal standard variate for significant level as identified in previous section i.e normal standard variate at 95% confidence level = 1.96. P1-P2 = different in proportions or effect size identified from previous studies. P1 = is proportion in cases (subjects) (0.17) [13]. P2 = is proportion in controls (0.04) [13]. n = minimum sample size.


$$n=\frac{1+1}{1}\times \frac{(0.5)(1-0.5){\left(0.84+1.96\right)}^{2}}{{\left(0.17-0.04\right)}^{2}}=232$$

Furthermore, a second formula correcting the sample size for a finite population was used because the first formula was drawn from a finite population of HIV-infected children numbering less than 10,000 population, thus:


$$nf=\frac{n}{1+(n/n_{E})}$$

Where, nf = represent sample size for a finite population n = represent a population greater than 10,000 participants. nE = estimated total number of HIV-infected males aged 8-17 years in FETHA and Mile Four Hospital were approximately 105 (61+44). Hence;


$$nf=\frac{232}{1+(232/105)}=73$$

The minimum sample size was thus 73.

### Non-responders rate


$$n\times \frac{10}{100}=7.3$$

Therefore, the actual minimum sample size of cases (subjects) was 80. However, the sample size is allocated proportionally to the two hospitals under study thus: let N_1_be the number of HIV patient aged 8-17 years in FETHA and N_2_be the number of HIV patient aged 8-17 years mile four, n = minimum sample size = 80 and N = total number of HIV patient aged 8-17 years in the two hospital = 105. Therefore, the sample size for each hospital is given by: for FETHA;


$$n_{1}=\frac{n\times N_{1}}{N}=\frac{80\times 61}{105}=46=57.5\%$$

For mile four;


$$n_{2}=\frac{n\times N_{2}}{N}=\frac{80\times 44}{105}=34=42.5\%$$

The same proportion was used for selection of the control.

**Sampling method:** perinatally HIV-infected males (subjects) who met the inclusion criteria were enrolled consecutively during the weekly paediatric HIV clinics. On enrollment, informed consent from the parents/caregivers and assent from the subjects were obtained by the researchers. Oyedeji social classification system was used to determine the social class of each participants. It was classified into high, medium and low based on the occupation and level of education of the parents. The social class is graded on a score of 1 to 5, where 1 being the highest and 5 being the lowest. Each score has two variables, occupation and level of education. The social class was further categorised into low (scores 4-5), middle (scores 2-3) and upper (scores 1-2) socio-economic class. Baseline World Health Organization (WHO) clinical staging and immunological classification of subjects on enrollment into the paediatric HIV clinic was obtained from the children´s case record forms. The WHO clinical staging was based on the WHO case definitions for HIV surveillance and clinical staging in children [14]. Standardized clinical parameters based solely on patients´ clinical features were used to delinate patients into different stages from stage 1 (asymptomatic) to stage 4 (AIDS) [14]. The HIV-uninfected males (controls) were enrolled from the Children Outpatient clinics after informed consent from caregivers and assent from the controls had been obtained by the researchers. The controls received HIV counseling and testing (HCT), according to national algorithm. The HIV negative controls, were subsequently enrolled and matched for age and social class with the subjects. A case record form was then completed for them by the researchers.

The subjects and the controls were examined by one of the researchers in a well-lit private room with a male chaperone. When private examination rooms were unavailable, privacy was ensured by using portable screening curtains to provide privacy for the participants. Tanner staging was identified, using the standardized method proposed by Marshall and Tanner for staging of sexual maturation [2]. The staging was done by examination of testicular volume and pubic hair growth. Pubic hair development was also staged from one to five. In the pre-pubertal stage 1, there was no pubic hair. Stage 2 was characterized by scanty, long and slightly pigmented hair while in stage 3, the hair is darker, starting to curl but in small amounts. In stage 4, pubic hair resembled adult pubic hair but less in quantity, coarse and curly and finally in stage 5, the pubic hair increases further in volume, spreads onto the medial thighs Estimation of testicular volume was done using an orchidometer. The right testis was first examined before the left testis for consistency and uniformity of data collection. Placing the participants in supine and cross-legged position, the testis was gently isolated. Then without squeezing the testis, scrotal skin was stretched. A manual side by side comparison between the testis and beads was made to establish the bead whose size matches that of the testis using a Prader orchidometer. Stage 1 is when testicular volume is less than 4 ml, stage 2 is 4-8 ml, stage 3 is 9-12 ml, stage 4 is 15-20 ml and stage 5 is when testicular volume is greater than 20 ml [13]. Puberty were classified as having started if they were at Tanner stage II or greater for pubic hair development [2]. Pubic hair development was classified as delayed puberty if pubic hair had not started by age 14 years [5,15]. All information gathered were documented in the case record form.

**Ethical consideration:** ethical approval was obtained from the Health Research and Ethics Committee of the Alex Ekwueme Federal University Teaching Hospital (FETHA/REC/VOL1/2017/597) and permission letter from Mile Four Mission Hospital (RE/M4H/30/18). The confidentiality of information and data retrieved from the study participants were emphasized to all study participants and were kept throughout and after the study. All the essential data relating to the survey were kept safe by the principal investigator. The study participants were assigned identification numbers and their data coded using their initials and identification numbers.

**Data analysis:** data were analyzed, using the Statistical Package for Social Sciences (SPSS) version 20 for windows (IBM Corp., Armonk, NY, USA). Descriptive statistics (frequencies, percentages, means and standard deviations) were generated for the variables. Means of continuous variables were compared, using the Student´s t-test, while effect of HIV status on testicular volume/pubic hair development were evaluated using logistic regression. All tests were two-tailed and conducted at 5% level of significance.

## Results

A total of 80 HIV-infected subjects and 80 HIV uninfected controls within the same age bracket of 8-17 years were enrolled for the study and matched for age and socio-economic class. Majority of the subjects and controls (70%) were pre/early adolescent (8-14 years) stage of life while 30% of the study participants were in middle adolescent (15-17 years). Forty-eight of the subjects (60%) and 48 controls (60%) respectively were in the lower socio-economic class while 32 (40%) were in the upper socio-economic class but none of the participants were from middle class. Table 1 is the clinical characteristics of the subjects. It shows that more of the subjects 64 (80%) were on first line of HAART. The table also depicts that 3 (3.75%) subjects were on HAART for less than 6 months while majority of them had been on these drugs for more than 5 years. In clinical staging of the subjects, 22 (27.5%) of them were in stage 3 while 1 (1.25%) subject was in stage 4. Additionally, 43 subjects (55.8%) had unsuppressed viral load while 34 (44.2%) had suppressed viral load. Three (3) subjects were on HAART for less than 6 months and were not classified as suppressed or unsuppressed viral load.

**Table 1:** clinical characteristics of the subjects

Characteristics	Categories	Frequency	Percent
ART regimen	First line	64	80.0
	Second line	16	20.0
Duration on ART	< 6 months	3	3.75
	6 months - 5 years	36	45.00
	> 5 years	41	51.25
Clinical stages	1	38	47.50
	2	19	23.75
	3	22	27.50
	4	1	1.25
Immunological classification (CD4 count)	Not significant	49	61.25
	Mild	14	17.50
	Advanced	12	15.00
		5	6.25
**Total**	Severe	80	100
Viral load	Suppressed	34	44.2
		43	55.8
**Total**	Unsuppressed	77	100

Not significant: >500ml/mm^3^, Mild: (350-499) ml/mm^3^, Advanced: (200-349) ml/mm^3^, Severe: < 200ml/mm^3^. Suppressed: ≤ 1000 copies/ml, Unsuppressed: > 1000 copies/ml

**Secondary sexual characteristics of the study groups:** the age distribution and testicular development of the study participant are shown in Table 2. Thirty-seven (46.25%) subjects compared to 7 (8.75%) controls were still in pre-pubertal stage while seventy-three (91.25%) controls compared to forty-three (53.75%) subjects were in pubertal stage of testicular development. The mean testicular volume for the subjects (8.288±8.259) was significantly lower than the controls (11.575±8.717) with p-value of 0.015. Table 3 depicts the age distribution and pubic hair development of the study participants. Forty-five (56.25%) of the subjects were in the pre-pubertal stage of pubic hair development, when compared with 27 (33.75%) of the controls, (p-value <0.001). For pubertal pubic hair development, 53 (66.25%) of the controls were in pubertal growth while only 35 (43.75%) of the subjects were pubic hair development with p-value of <0.001 as shown in Table 3.

**Table 2:** age distribution and testicular size development of the study participants

Age (years)	Testicular size					
	Pre-pubertal (<4ml)			Pubertal (≥4ml)		
	Subjects (%)	Controls (%)	P-value	Subjects (%)	Controls (%)	P-value
8 9 10 11 12 13 14 15 16 17	10(12.50)	6(7.50)	0.2488	0(0.0)	4(5.00)	0.0235
	9(11.25)	0(0.00)	0.0006	1(1.25)	10(12.50)	0.0009
	9(11.25)	1(1.25)	0.0029	2(2.50)	10(12.50)	0.0091
	5(6.25)	0(0.00)	0.0129	2(2.50)	7(8.75)	0.0370
	4(5.00)	0(0.00)	0.0235	4(5.00)	8(10.00)	0.0897
	0(0.00)	0(0.00)	-	3(3.75)	3(3.75)	-
	0(0.00)	0(0.00)	-	7(8.75)	7(8.75)	-
	0(0.00)	0(0.00)	-	5(6.25)	5(6.25)	-
	0(0.00)	0(0.00)	-	7(8.75)	7(8.75)	-
	0(0.00)	0(0.00)	-	12(15.00)	12(15.00)	-
**Total**	37(46.25)	7(8.75)	<0.0001	43(53.75)	73(91.25)	<0.0001

Subjects= HIV-positive, controls= HIV-negative

**Table 3:** age distribution and pubic hair development of the study participants

Age (years)	Pubic hair					
	Pre-pubertal			Pubertal		
	Subjects (%)	Controls (%)	P-value	Subjects (%)	Controls (%)	P-value
8	10(12.50)	9(11.25)	0.807	0(0)	1(1.25)	0.157
9	10(12.50)	7(8.75)	0.441	0(0)	3(3.75)	0.040
10	10(12.50)	4(5.00)	0.045	1(1.25)	7(8.75)	0.015
11	6(7.50)	3(3.75)	0.151	1(1.25)	4(5.00)	0.086
12	5(6.25)	4(5.00)	0.366	3(3.75)	4(5.00)	0.350
13	1(1.25)	0(0.00)	0.157	2(2.50)	3(3.75)	0.325
14	1(1.25)	0(0.00)	0.157	6(7.50)	7(8.75)	0.386
15	2(2.50)	0(0.00)	0.076	3(3.75)	5(6.25)	0.234
16	0(0.00)	0(0.00)	-	7(8.75)	7(8.75)	-
17	0(0.00)	0(0.00)	-	12(15.00)	12(15.00)	-
Total	45(56.25)	27(33.75)	<0.001	35(43.75)	53(66.25)	<0.001

Subjects=HIV-positive, controls=HIV-negative

**Assessment of the pattern of secondary sexual maturation of the study groups:** most of the subjects were in pre-pubertal stages for testicular development and pubic hair development [TSI=37 (46.25%) and PH1=45 (56.25%)] compared to the controls [TS1=7 (8.75%) and PHI=27 (33.75%)] as shown in Table 4. The controls were about twice more likely to attain puberty than subjects (p = 0.005, OR=2.524, CI=1.330-4.787). Controls were more likely to achieve puberty by testicular development than subjects with p-value <0.001, OR=8.973, CI=3.680-21.882.

**Table 4:** sexual maturation of the study participants

Variable	Participants		OR	P-value	95% C.I. for OR
	Subjects (n=80)	Controls (n=80)			
**Testicular size**					
Stage 1	37(46.3)	7(8.8)	13.592	<0.001	4.137-44.653
Stage 2	17(21.3)	38(7.5)	1.150	0.792	0.405-3.267
Stage 3	4(5.0)	6(7.5)	1.714	0.492	0.369-7.974
Stage 4	15(18.8)	11(13.8)	3.506	0.035	1.089-11.291
Stage 5 (ref.)	7(5.0)	18(22.5)	1		
**Public hair**					
Stage 1	45(56.3)	27(33.8)	0.425	0.032	0.194-0.930
Stage 2	12(15.0)	17(21.3)	1.003	0.994	0.382-2.635
Stage 3	4(5.0)	7(8.8)	1.240	0.760	0.313-4.911
Stage 4	2(2.5)	5(6.3)	1.771	0.523	0.307-10.227
Stage 5 (ref.)	17(21.3)	24(30.0)	1		

The reference category (ref.) for each variable is the last category. OR =odd ratio, CI confidence interval, subjects=HIV-positive, Controls=HIV-negative. The odds ratio used was unadjusted OR because the two variables under consideration (testicular size and pubic hair) are both proxy for sexual maturation. Both should not be included in the same model to avoid “multicollinearity”

**Examining relationship between sexual maturation and some modifiable factors:** Table 5 is the result of the logistic regression of sexual maturation (testicular size and pubic hair) on some modifiable factors (CD count, clinical stages and duration on HAART). The result showed a significant relationship between sexual maturation and clinical stages. It further showed an odd ratio of less than 1 in all the categories of the clinical stages, indicating a better sexual maturation in each of those stages compare to the reference category (stage IV). Further, the result showed a significant relationship between pubic hair growth with duration on HAART.

**Table 5:** regression analysis of sexual maturation on social class, immunological stage, clinical stage and duration on HAART

Variable	Testicular size				Pubic hair			
	β	AOR	Wald statistic	P-value	β	AOR	Wald statistic	P-value
Intercept	-5.359	0.005	46.667	<0.001	-5.217	0.005	41.934	<0.001
**Social class**								
Lower	0.337	1.401	1.186	0.276	0.341	1.406	1.105	0.293
**Upper (ref.)**								
**CD 4 count**								
Not significant	-0.761	0.467	0.962	0.327	-0.892	0.410	1.241	0.265
Mild	-0.221	0.802	0.074	0.786	-0.427	0.652	0.259	0.610
Advance	0.108	1.114	0.016	0.900	-0.039	0.962	0.002	0.964
Severe (ref.)		1				1		
**Clinical stage**								
Stage I	-5.422	0.004	217.49	<0.001	-5.908	0.003	243.79	<0.001
Stage II	-5.304	0.005	154.62	<0.001	-5.407	0.004	159.02	<0.001
Stage III	-5.093	0.006	108	0.043	-5.187	0.006	124	0.038
Stage IV (ref.)		1				1		
Duration on HAART	0.057	1.059	1.695	0.193	0.103	1.108	5.251	0.022

p<0.05 indicates significant coefficient, β = value of regression coefficient, Wald= Wald statistics for test of significant of regression coefficient, the last category for each categorical variable is reference category (ref.), AOR= adjusted odds ratio An adjusted odds ratio was adopted because each of the independent variables are linearly uncorrelated (i.e. there is no multicollinearity), hence the regression model is multiple regression model.

## Discussion

HIV infection was noted to negatively affect the total number of males that attained sexual maturation in the index study. More of the subjects were in pre-pubertal stage of testicular development 37 (46.25%) than controls 7 (8.75%). In subsequent testicular development (TS II), (21.25%) of the subjects were in stage II compared with controls (47.5%) on the same stage. Therefore, HIV uninfected controls were observed to develop faster from testicular stage II to stage V more than the HIV-infected subjects. This implies that the subjects were more likely to be in the pre-pubertal stage using testicular size development, which leads to delayed pubertal development in the subjects. This may be due to disruption of the gonadotropin-releasing hormone axis. Ebling FJ and Ojeda SR *et al*. [16,17], observed that pulsatile secretion of GnRH helps in the pituitary secretion of luteinizing hormone (LH) and follicle-stimulating hormone (FSH) in males, which helps in testicular development and spermatogenesis respectively. This pathway is disrupted in HIV-infection and subsequently affects pubertal development. Puberty begins with testicular enlargement to greater than 2.5cm in length or greater than 4mls in volume initiated by pulsatile secretion of GnRH from its neuron and therefore delay in pubertal development is often a function of testicular development. A study by Majaliwa ES *et al*. [10] found that there is delay in pubertal development in prenatally HIV-infected children. This study agrees with the findings by Majaliwa ES *et al*. [10].

However, Kessler M *et al*. [18] found no difference in testicular development between subject and control. Also, the age of onset of tanner stage 4 was similar in both groups in their study. Furthermore, in this study, more subjects were also noted to be in the pre-pubertal stage of pubic hair development 45 (56.25%) than the controls 27 (33.75%). This is to be expected since in majority of adolescents testicular development preceded pubic hair. Therefore, delay in testicular development will likely lead to delay in development of pubic hair. Williams PL *et al*. [19] noted that for both subjects and controls testicular development preceded pubic hair development. These findings imply that HIV infection is associated with delayed initiation and subsequent progression through pubertal stages of testicular and pubic hair development.

Similar findings were found on the pubic hair development indicating that subjects were more likely to remain in the pre/early adolescent period than the controls. The difference in age compared favorably with the findings made by Mbwile GR [20] in Tanzania and Williams PL *et al*. [19] in the United States. The explanation for the age difference in HIV-infected versus HIV uninfected children may be due to the general effects of chronic illness as demonstrated by Pozo J and Argente J [21]. The study by Buchacz K *et al*. [11] corroborated well with this study and showed that there was delayed onset of pubertal development in children and adolescent with HIV infection. In addition, the studies by Buchacz K *et al*., Mbwile GR, Pozo J & Argente J and Iloh ON *et al*. [11,20-22] found that HIV infection affected the onset of puberty and progression into different pubertal stages. This suggests that HIV infection is associated with delayed onset and progression through the pubertal stages of testicular size and pubic hair development in HIV-infected children and adolescents. HIV-infected males were also observed to be less likely to reach PH2-PH5 which indicates the delaying progression to full sexual maturity and initiation than the HIV-uninfected controls. These observations further reiterate the fact that HIV infection may lead to delay in both the timing of initiation of puberty and the progression through other subsequent pubertal stages [4]. Similar findings were documented by Mbwile GR [20] and Szubert AJ *et al*. [23] in Africa and de Martino M *et al*. [24] in Italy. These buttress the fact that HIV infection probably because of the chronicity of the disease and opportunistic infections (OIs) that they are prone to may negatively affects the age of pubertal development.

There was significant relationship between CD4 count and development of pubic hair. This suggests that subjects in advanced or in a severe immunologically depressed stage may have delay in attainment of puberty in HIV-infected children. The reason for the observation may be because subjects who have low CD4 count are prone to opportunistic infection which may lead to poor growth. Bellavia A *et al*. [25] observed that there was delay in sexual maturation in HIV-infected children which resulted from poor growth. The observation of delayed pubic hair development in subjects who have low CD4 count is supported by de Martino M *et al*. [24] and Buchacz K *et al*. [11] who in their studies noted that delayed puberty is more common in children with low CD4 count.

The logistic regression analysis of the present study showed that clinical stages was significantly related to the sexual maturation. This may be explained by the facts that opportunistic infections may affect the pubertal development and sexual maturation. Furthermore, there was a positive significant relationship between duration on HAART and sexual maturation. This may be explained by the facts that ARV drugs may lead to restoration of immunity and subsequently improvement in sexual maturation. The study by Mbwile GR [20] supported this finding and noted that ARVs helps in improvement in sexual development and maturation. Szubert AJ *et al*. [23] observed that early initiation of HAART will lead to males attaining sexual maturity in earlier age than those who started ARV drugs at an older age. Williams PL *et al*. [19] also supported the fact that ART may result in more normal pubertal onset and development.

## Conclusion

There was a significant delay in both the age at onset and progression of sexual maturation in HIV-infected subjects compared to their HIV-uninfected counterparts.

**Limitation:** the study was a case-control hospital-based study with only one assessment of physical growth and sexual maturation. A prospective cohort study with multiple interval assessments would have given more information on the age at onset of testicular enlargement and pubic hair development as well as the subsequent pubertal development of HIV-infected males.

### What is known about this topic

- *Adolescents infected vertically by HIV are at higher risk of developmental impairment, growth alteration, wasting, delayed- puberty and impaired neuro-cognitive function;*
- *An actual pubertal delay in sexual maturation in adolescent can lead to the development of poor body image and low self-esteem which may result in psychosocial problems like school avoidance, poor academic achievement, isolation, eating disorders, bullying/teasing by peers, depression, and social withdrawal*.

### What this study adds

- *This study affirms that there is a significant delay in both the age at onset and progression of sexual maturation in HIV-infected subjects compared to their HIV-uninfected counterparts;*
- *There was an association between HIV disease severity and delay in sexual maturation*.
